# Supplementary material for: Lower cancer incidence three years after COVID-19 infection in a large veteran population
Source: PLoS One. 2025 Aug 25;20(8):e0318131. doi: 10.1371/journal.pone.0318131 (PMC12377599; doi:10.1371/journal.pone.0318131)
Supplement: Attachment — Submitted filename: Response to Reviews.docx [file pone.0318131.s003.docx]

**Miami, June 04^th^, 2025**

Anna D. Ware, MPH, MS
Academic Editor
PLOS ONE

**RE: Resubmission to PLOS ONE, PONE-D-24-59823**

Dear Ms. Ware,

**Thank you again for reviewing our manuscript entitled “Lower Cancer Incidence Three Years After COVID-19 Infection in a Large Veteran Population ”, which we would like to resubmit as an original article in PLOS ONE. We appreciate the reviewers’ comments and feedback and have edited the manuscript according to their suggestions. We have made the changes to the documents using the tracked changes feature, and a clean copy without tracking has also been included. Line item changes referenced in our responses correspond to the “clean manuscript”.**

**This manuscript has not been previously published and is not under review by another journal. All listed authors qualify for authorship, and all those who qualify as authors are listed. My co-authors and I believe it is a valuable submission to the PLOS ONE.**

**On behalf of my co-authors and myself, we are looking forward to your response.**

Respectfully,

Iriana S. Hammel MD, FACP, AGSF

Program Director, University of Miami/Jackson Health System Geriatric Medicine Fellowship

Assistant Professor, Geriatric Medicine, University of Miami, Miller School of Medicine

Member, Global Frailty Network

Associate Director for Education and Evaluation,

Geriatric Research, Education and Clinical Center (GRECC)

Bruce W. Carter Miami VAMC

Telephone: (305)575-3388

E-mail: [iriana.hammel@med.miami.edu](mailto:iriana.hammel@med.miami.edu)

**Journal Requirements:**

When submitting your revision, we need you to address these additional requirements.

**1. Please ensure that your manuscript meets PLOS ONE's style requirements, including those for file naming. The PLOS ONE style templates can be found at**

[https://journals.plos.org/plosone/s/file?id=wjVg/PLOSOne_formatting_sample_main_body.pdf](https://gcc02.safelinks.protection.outlook.com/?url=https%3A%2F%2Furldefense.com%2Fv3%2F__https%3A%2Ftrack.editorialmanager.com%2FCL0%2Fhttps%3A*2F*2Fjournals.plos.org*2Fplosone*2Fs*2Ffile*3Fid%3DwjVg*2FPLOSOne_formatting_sample_main_body.pdf%2F1%2F010f019687639e9b-53678831-2027-42f2-bebb-2c00acf21af9-000000%2F8qIT-Mn1lfDRc-LEq-tYvR24gEiuSzhxWfnVEz-UwuI%3D208__%3BJSUlJSUlJQ!!KVu0SnhVq1hAFvslES2Y!OgqCtDiZmC-hfchBETXS9gNJV798T7dcHR4rjpxsb67JNCVO_SIRGz4g1J91KCu-yhU20ne6zjQi3L-baK8A71k%24&data=05%7C02%7C%7Cd43b391d24c7419e9a5908dd881c7ec8%7Ce95f1b23abaf45ee821db7ab251ab3bf%7C0%7C0%7C638816378517636003%7CUnknown%7CTWFpbGZsb3d8eyJFbXB0eU1hcGkiOnRydWUsIlYiOiIwLjAuMDAwMCIsIlAiOiJXaW4zMiIsIkFOIjoiTWFpbCIsIldUIjoyfQ%3D%3D%7C0%7C%7C%7C&sdata=Wl6fcatIsUJb%2BMkahNfQO%2B4ljgbLyCQR3hpgi%2FIKJck%3D&reserved=0) and

[https://journals.plos.org/plosone/s/file?id=ba62/PLOSOne_formatting_sample_title_authors_affiliations.pdf](https://gcc02.safelinks.protection.outlook.com/?url=https%3A%2F%2Furldefense.com%2Fv3%2F__https%3A%2Ftrack.editorialmanager.com%2FCL0%2Fhttps%3A*2F*2Fjournals.plos.org*2Fplosone*2Fs*2Ffile*3Fid%3Dba62*2FPLOSOne_formatting_sample_title_authors_affiliations.pdf%2F1%2F010f019687639e9b-53678831-2027-42f2-bebb-2c00acf21af9-000000%2FJDqf3DNF7Zx3aQ9lKZy2d8xG3Rz9rG4Wd78DLDA6Jbs%3D208__%3BJSUlJSUlJQ!!KVu0SnhVq1hAFvslES2Y!OgqCtDiZmC-hfchBETXS9gNJV798T7dcHR4rjpxsb67JNCVO_SIRGz4g1J91KCu-yhU20ne6zjQi3L-b-ho4EZw%24&data=05%7C02%7C%7Cd43b391d24c7419e9a5908dd881c7ec8%7Ce95f1b23abaf45ee821db7ab251ab3bf%7C0%7C0%7C638816378517649647%7CUnknown%7CTWFpbGZsb3d8eyJFbXB0eU1hcGkiOnRydWUsIlYiOiIwLjAuMDAwMCIsIlAiOiJXaW4zMiIsIkFOIjoiTWFpbCIsIldUIjoyfQ%3D%3D%7C0%7C%7C%7C&sdata=IRje8BE3QbjrU3UfeQbMCfmz4f7rB9aLxpoWR9uKMOE%3D&reserved=0)

**2. Thank you for stating the following in the Acknowledgments Section of your manuscript:**

“This study was supported using resources and facilities of VINCI, VA HSR RES 13-457”

We note that you have provided additional information within the Acknowledgements Section that is not currently declared in your Funding Statement. Please note that funding information should not appear in the Acknowledgments section or other areas of your manuscript. We will only publish funding information present in the Funding Statement section of the online submission form.

Please remove any funding-related text from the manuscript and let us know how you would like to update your Funding Statement. Currently, your Funding Statement reads as follows:

“This study was supported using resources and facilities of VINCI, VA HSR RES 13-457. The funder did not play any role in the study design, data collection and analysis, decision to publish or preparation of the manuscript.”

**Response:** This study was supported by VINCI but not funded. Therefore, the funding statement has been removed. VINCI has been added to the acknowledgement section.

***Added Line 313-315: “***We thank VINCI for their database that was provided to us, under VA Health Services Research Office No. 13-457. RES 13-457”

**3. We note that the grant information you provided in the ‘Funding Information’ and ‘Financial Disclosure’ sections do not match.**

When you resubmit, please ensure that you provide the correct grant numbers for the awards you received for your study in the ‘Funding Information’ section

**Response:** There was no funding for this project.

**4. Please include your full ethics statement in the ‘Methods’ section of your manuscript file. In your statement, please include the full name of the IRB or ethics committee who approved or waived your study, as well as whether or not you obtained informed written or verbal consent. If consent was waived for your study, please include this information in your statement as well.**

**Response: Ethics statement added (Line 78-80):** “The study design and protocol were approved by the Miami VA Healthcare System Research and Development Committee (Reference: 1592780-10). Informed consent was waived as this is a data only research project.”

**5. We note that you have indicated that there are restrictions to data sharing for this study. PLOS only allows data to be available upon request if there are legal or ethical restrictions on sharing data publicly. For more information on unacceptable data access restrictions, please see** [http://journals.plos.org/plosone/s/data-availability#loc-unacceptable-data-access-restrictions](https://gcc02.safelinks.protection.outlook.com/?url=https%3A%2F%2Furldefense.com%2Fv3%2F__http%3A%2Ftrack.editorialmanager.com%2FCL0%2Fhttp%3A*2F*2Fjournals.plos.org*2Fplosone*2Fs*2Fdata-availability*23loc-unacceptable-data-access-restrictions%2F1%2F010f019687639e9b-53678831-2027-42f2-bebb-2c00acf21af9-000000%2FIBzW7AS21lh7UsJlirDFCTgfWrYFgzQRu2x_egHLzXc%3D208__%3BJSUlJSUl!!KVu0SnhVq1hAFvslES2Y!OgqCtDiZmC-hfchBETXS9gNJV798T7dcHR4rjpxsb67JNCVO_SIRGz4g1J91KCu-yhU20ne6zjQi3L-bV-5mZ30%24&data=05%7C02%7C%7Cd43b391d24c7419e9a5908dd881c7ec8%7Ce95f1b23abaf45ee821db7ab251ab3bf%7C0%7C0%7C638816378517663158%7CUnknown%7CTWFpbGZsb3d8eyJFbXB0eU1hcGkiOnRydWUsIlYiOiIwLjAuMDAwMCIsIlAiOiJXaW4zMiIsIkFOIjoiTWFpbCIsIldUIjoyfQ%3D%3D%7C0%7C%7C%7C&sdata=j5kLITATIIBUkEHVqnlmp7ZSrz1aFusVwPRq%2Fy1gT1s%3D&reserved=0).

Before we proceed with your manuscript, please address the following prompts:

If there are ethical or legal restrictions on sharing a de-identified data set, please explain them in detail (e.g., data contain potentially identifying or sensitive patient information, data are owned by a third-party organization, etc.) and who has imposed them (e.g., a Research Ethics Committee or Institutional Review Board, etc.). Please also provide contact information for a data access committee, ethics committee, or other institutional body to which data requests may be sent.

**Response**: We are not able to share the data directly as we obtained access to the U.S. VA national data through VA Informatics and Computing Infrastructure (VINCI). We signed an agreement that the data will not be downloaded and will stay at the VINCI Workspaces behind VA firewall. Any request for the data can be sent to VINCI at [VINCI@va.gov](mailto:VINCI@va.gov).

**Additional Editor Comments:**


Thank you for your valuable contributions to this important topic, which holds significant relevance for the scientific and clinical communities. Your study provides a meaningful evaluation of the association between COVID-19 infection and cancer incidence among Veterans, offering timely insights into a complex and evolving issue. Before we can proceed toward acceptance, the reviewers have suggested a major revision. Please find their feedback attached. In addition, I have outlined several suggestions below that I believe would help strengthen the clarity and robustness of your manuscript:

**1. Please provide a reference supporting the dates you assign to the Alpha and Beta variant waves (March 15–November 30, 2020).**

**Response**: The first strain of COVID-19 (L-strain) was identified in Wuhan, China, in Dec 2019. The first variants of interest weren’t recognized until end of 2020.* Given the timeframe we used for our study, from March to November 2020, most of our cases are likely the original COVID-19 L-strain. We have removed the terms Alpha and Beta variants from our manuscript.

**Removed Line 69:** (Alpha/Beta)

**Added Line 68-69:** This time period encompasses the initial strain of COVID-19, before the emergence of the alpha and beta waves in December of 2020(10, 11)

**Additional References**

Alpha (B.1.1.7) – UK Sept 2020: “The modeled trajectory of this variant in the U.S. exhibits rapid growth in early 2021, becoming the predominant variant in March” **

Beta (B.1.351) – South Africa end of 2020: “As of January 12, 2021, neither the B.1.351 nor the P.1 variants have been detected in the United States” **

* Historical working definitions and primary actions for SARS-CoV-2 variants. WHO.

**Galloway SE, Paul P, MacCannell DR, et al. Emergence of SARS-CoV-2 B.1.1.7 Lineage — United States, December 29, 2020–January 12, 2021. MMWR Morb Mortal Wkly Rep 2021;70:95–99. DOI: <http://dx.doi.org/10.15585/mmwr.mm7003e2>

Chatterjee S, Bhattacharya M, Nag S, Dhama K, Chakraborty C. A Detailed Overview of SARS-CoV-2 Omicron: Its Sub-Variants, Mutations and Pathophysiology, Clinical Characteristics, Immunological Landscape, Immune Escape, and Therapies. Viruses. 2023 Jan 5;15(1):167. doi: 10.3390/v15010167. PMID: 36680207; PMCID: PMC9866114.

**2. Consider separating Alpha and Beta wave periods if possible, as different variants are associated with differing clinical outcomes.**

**Response**: Our study period occurs before the emergence of the alpha and beta waves within the US and we corrected this in the paper.

**3. Clarify how subsequent infections were handled. If a patient had multiple positive tests, which infection date was used to determine exclusion and outcomes?**

**Response:** We were unable to account for re-infection data due to the following reasons, which we added to the discussion section of the manuscript, as below:

**Added Line 286 – 298:** “We attempted to account for re-infection data; however, we were unable to capture all cases due to the increased availability of COVID-19 testing outside of the VA, such as at-home testing, state, and county testing sites, among others, and during subsequent re-infections. Veterans who may have had milder reinfection and did not seek treatment or testing, or other medical and socioeconomic factors, which may have limited testing, could not be accounted for, beyond the initial study window. It is unknown how reinfection alters the risk of cancer development in these patients, and the extent to which it might induce other physiological changes. For our study, we separated the groups based on an initial index infection in which a veteran either tested positive or negative for COVID-19. Veterans were maintained in this initial grouping for the three-year follow-up period, even if those in the initial COVID-19 negative group may have subsequently developed an infection at a later period during the follow-up window or those in the initial group had reinfection. As a result, our findings may not accurately reflect the true effect size of this association.”

**4. Expand on how “active patient in the past 12 months” was defined (e.g., minimum number of visits or types of care). Also, revise the description to avoid run-on structure**.

## **Response**:

## Please see below the description of the “active patients in the past 12 months” that was added to the Study Design, Data Sources, and Study Population in the Methods section of the manuscript:

**Added Line 72-73** “and those who were not active patients in the past 12 months (active were defined as having at least one visit to a VA primary care clinic)”

**5. Describe how subsequent infections were treated in your time-to-event analyses beginning 30 days post-infection.**

**Response**: We were unable to account for re-infection data due to the following reasons, which we added to Discussion section of the manuscript, as below:

**Added Line 286 – 298** “We attempted to account for re-infection data; however, we were unable to capture all cases due to the increased availability of COVID-19 testing outside of the VA, such as at-home testing, state, and county testing sites, among others, and during subsequent re-infections. Veterans who may have had milder reinfection and did not seek treatment or testing, or other medical and socioeconomic factors, which may have limited testing, could not be accounted for, beyond the initial study window. It is unknown how reinfection alters the risk of cancer development in these patients, and the extent to which it might induce other physiological changes. For our study, we separated the groups based on an initial index infection in which a veteran either tested positive or negative for COVID-19. Veterans were maintained in this initial grouping for the three-year follow-up period, even if those in the initial COVID-19 negative group may have subsequently developed an infection at a later period during the follow-up window or those in the initial group had reinfection. As a result, our findings may not accurately reflect the true effect size of this association.”

**6. Include model fit statistics (e.g., AIC, C-index, Schoenfeld residuals) to strengthen the robustness of your Cox model results.**

**Response**: We added the C-index to the manuscript.  We chose to present the C-index instead of using AIC as all the covariates in the model were chosen based on their clinical relevance and not from a model selection perspective.

We added to the subsection “Association of COVID-19 Infection and Decreased Cancer Risk”:

Line 119-120: “The C-Index for the model was 0.733 (se=0.001), indicating moderate predictive accuracy.”

We mentioned in the method that:

**Line 98-100:** “The proportional hazard ratio assumption was assessed and verified by testing the correlation between the Schoenfeld residuals and survival time.”

**7. Reconsider whether “active patient status” should be an exclusion criterion rather than a covariate, given its implications for care utilization and potential external healthcare encounters.**

**Response:** We agree with the reviewer’s comment, and we excluded patients who were not active users of the VA system . We have re-done the analysis. Please see Results and Tables1-6. We added to the Method section about this exclusion criterion:

Line 72-73: “….and those who were not active patients in the past 12 months defined as having at least one or more visits to a VA primary care clinic.”

**Reviewers' comments:

Reviewer's Responses to Questions**

**Comments to the Author**

1. Is the manuscript technically sound, and do the data support the conclusions?

The manuscript must describe a technically sound piece of scientific research with data that supports the conclusions. Experiments must have been conducted rigorously, with appropriate controls, replication, and sample sizes. The conclusions must be drawn appropriately based on the data presented.

Reviewer #1: Partly

Reviewer #2: Yes

2. Has the statistical analysis been performed appropriately and rigorously?

Reviewer #1: Yes

Reviewer #2: Yes

3. Have the authors made all data underlying the findings in their manuscript fully available?

The [PLOS Data policy](https://gcc02.safelinks.protection.outlook.com/?url=https%3A%2F%2Furldefense.com%2Fv3%2F__http%3A%2Ftrack.editorialmanager.com%2FCL0%2Fhttp%3A*2F*2Fwww.plosone.org*2Fstatic*2Fpolicies.action*23sharing%2F1%2F010f019687639e9b-53678831-2027-42f2-bebb-2c00acf21af9-000000%2FeKuDnC3mKudMttdpHngNVmjFI1aFgJIj5OY_dA4WilU%3D208__%3BJSUlJSU!!KVu0SnhVq1hAFvslES2Y!OgqCtDiZmC-hfchBETXS9gNJV798T7dcHR4rjpxsb67JNCVO_SIRGz4g1J91KCu-yhU20ne6zjQi3L-bq20nw0w%24&data=05%7C02%7C%7Cd43b391d24c7419e9a5908dd881c7ec8%7Ce95f1b23abaf45ee821db7ab251ab3bf%7C0%7C0%7C638816378517691166%7CUnknown%7CTWFpbGZsb3d8eyJFbXB0eU1hcGkiOnRydWUsIlYiOiIwLjAuMDAwMCIsIlAiOiJXaW4zMiIsIkFOIjoiTWFpbCIsIldUIjoyfQ%3D%3D%7C0%7C%7C%7C&sdata=42kRs00ljKdSvUpISZDkZkPKzfuw%2FzYAgZzFTAEclFM%3D&reserved=0) requires authors to make all data underlying the findings described in their manuscript fully available without restriction, with rare exception (please refer to the Data Availability Statement in the manuscript PDF file). The data should be provided as part of the manuscript or its supporting information, or deposited to a public repository. For example, in addition to summary statistics, the data points behind means, medians and variance measures should be available. If there are restrictions on publicly sharing data—e.g. participant privacy or use of data from a third party—those must be specified.

Reviewer #1: Yes

Reviewer #2: No

4. Is the manuscript presented in an intelligible fashion and written in standard English?

PLOS ONE does not copyedit accepted manuscripts, so the language in submitted articles must be clear, correct, and unambiguous. Any typographical or grammatical errors should be corrected at revision, so please note any specific errors here.

Reviewer #1: Yes

Reviewer #2: Yes

**5. Review Comments to the Author**

Please use the space provided to explain your answers to the questions above. You may also include additional comments for the author, including concerns about dual publication, research ethics, or publication ethics. (Please upload your review as an attachment if it exceeds 20,000 characters)

Reviewer #1: Of course there are three possibilities--increase, decrease or no effect. This is an interesting correlational finding, and even when you pull the confounds out a 25% difference remains, which is large enough to justify publication, but it could just be a statistical fluke.... particularly as all those people on the other side of the study had their immune systems stimulated by the COVID vaccine, as well as other corona and influenza viruses, so you are really positing this particular infection leads to a more robust protective response--including that of the vaccine.

**Please see below our revised Discussion regarding effect of vaccination:**

**Added Line 298-304:** “Regarding vaccination status, our initial study cohort was grouped prior to the availability of the COVID-19 vaccination and was maintained in this cohort status regardless of subsequent vaccination status during the follow-up period. It is uncertain how exposure from vaccination with mRNA might enhance or induce monocyte creation, similarly to that observed in the previously cited study (9). In this study, inducible monocytes were only created when exposed to COVID-19 ssRNA and not that of influenza ssRNA. Further research is required to define potential mechanisms.”

Liu X, Ren Z, Tan C, Núñez-Santana FL, Kelly ME, Yan Y, Sun H, Abdala-Valencia H, Yang W, Wu Q, Toyoda T, Milisav M, Casalino-Matsuda SM, Lecuona E, Cerier EJ, Heung LJ, Abazeed ME, Perlman H, Gao R, Chandel NS, Budinger GRS, Bharat A. Inducible CCR2+ nonclassical monocytes mediate the regression of cancer metastasis. J Clin Invest. 2024 Oct 1;134(22):e179527. doi: 10.1172/JCI179527. PMID: 39545417; PMCID: PMC11563681.

**Reviewer #2:** This study presents an intriguing and timely investigation utilizing a large-scale cohort design, albeit with a relatively brief follow-up period. The topic carries substantial clinical relevance, and with appropriate revisions, the manuscript's impact could be considerably enhanced.

**Methods**

**1) Study outcome:**
The assertion that "screening returned to baseline" requires more robust empirical validation. Please incorporate specific quantitative data regarding outpatient encounters, screening utilization rates, or diagnostic procedural volumes in the post-pandemic period to substantiate this important claim.

**Response**: Thank you for this point of clarification. Please see the rewritten section of the manuscript below which attempts to address this issue.

**Revised Line 211 – 229:** “COVID-19 has introduced several new healthcare disruptions in cancer surveillance. Cancer trends during the pandemic showed an initial reduction, followed by a general return to the pre-pandemic levels (14, 15). One study looking at cancer screening rates among breast, cervical, and colorectal cancers found that screening initially dropped off during the start of the pandemic but resumed by the end of 2020, before experiencing a decline in 2021(16). An additional study showed that cancer incidence rates returned to their pre-pandemic trends in 2021, but those numbers did not fully incorporate delayed diagnosis from 2020 (17). These changes were expected, given the drastic reduction in preventive services in the US (18). An analysis of trends in US cancer rates revealed the highest variability in breast, cervical, prostate, and colorectal cancers, likely driven by a reduction in surveillance screening (19). By 2023, breast and colorectal screening had improved from the pandemic-related declines, with cervical cancer screening remaining below pre-pandemic levels (20). Additionally, the study collected data on all cancer types beyond those that are routinely screened for in clinical practice. It is uncertain how changes in cancer screening as described above, impacted the detection and treatment of these other cancer types. For this study, patients were followed for three years after the initial index infection. This included periods described above where the rates of screening for some cancer types returned to pre-pandemic levels or remained below expectations. Because both cohorts were exposed to similar screening delays and recovery over this period, it is unlikely that the difference in cancer rates between these groups could be attributed to this factor.”

**Results**

**1)Line 101:**
There is a numerical formatting error in "45,5388." Please rectify this typographical inconsistency.

**Response**: Corrected, thank you.

**2)Table 1:**
Consider a more rigorous presentation of between-group differences. The inclusion of standardized mean differences (SMDs) would facilitate a more precise assessment of covariate balance following propensity matching or multivariate adjustment.

**Response**: We did not present the standard mean differences as all of the variables presented in Table 1 were categorical variables, except for Age.

**3)Association of COVID-19 Infection and Decreased Cancer Risk:**

Lines 107-113 (Table 2)
Drawing substantive conclusions without stratification by cancer type is methodologically problematic. Given that various malignancies exhibit distinct pathophysiological mechanisms and screening paradigms, cancer-specific subgroup analyses would significantly enhance the interpretability of your findings and potentially elucidate specific protective associations.

**Response:** We agree with the reviewer and therefore we included the following paragraphs in the Results section of the manuscript, along with Table 6, which showed the hazard ratio for the top 10 cancer types for men.

**Added Lines 178-182:**

**“Association of COVID-19 Infection and Decreased Cancer Risk by Cancer Type”**

The association of COVID-19 infection and different types of newly diagnosed cancer risk was accessed (Table 6). In all of the ten cancer types, we observed reduced hazard of having new cancer diagnosis in COVID-19 positive patients (Table 6)”

|  | **Number of events (%)** | **aHR (95% CI)** |
| --- | --- | --- |
| Prostate Cancer | 9037 (1.8%) | 0.81 (0.77-0.86) |
| Lung Cancer | 5594 (1.1%) | 0.63 (0.57-0.69) |
| Colorectal cancer | 2222 (0.4%) | 0.66 (0.58-0.76) |
| Kidney Cancer | 1851 (0.4%) | 0.80 (0.70-0.92) |
| Liver Cancer | 1801 (0.4%) | 0.69 (0.59-0.80) |
| Leukemias | 1261 (0.3%) | 0.84 (0.71-0.98) |
| Oral cancer | 1452 (0.3%) | 0.57 (0.48-0.68) |
| Pancreatic Cancer | 1271 (0.3%) | 0.71 (0.60-0.84) |
| Bladder Cancer | 2301 (0.5%) | 0.66 (0.58-0.75) |
| Melanoma | 2768 (0.6%) | 0.81 (0.72-0.90) |

**Table 6.** aHR for New Cancer Diagnosis within three years for Male Patients with Positive COVID-19 Testing Compared to Patients of Negative Testing by Cancer Types. Adjusted for age, race, ethnicity, sex, BMI, smoking, kidney disease, COPD, diabetes, hypertension, hyperlipidemia, obesity, chronic lung disease, PTSD, alcohol dependence, drug dependence, exposure to toxic substances, other viral infections, and immunodeficiency

**Added Discussion Lines 271-276**

“Our study examined the top ten cancer types in men to determine if there was a primary driver behind the overall reduction observed. Surprisingly, no single cancer type was found to be influencing these results. All cancers showed various levels of reduction. These findings suggest a possible shared biological mechanism that might allow the immune system to identify cancer cells regardless of type. Further research is required to confirm the existence of this mechanism and how it influences the development of cancer.”

**4)Association of COVID-19 Infection and Decreased Cancer Risk by Age Groups, Gender, and Race: Lines 120-130 (Table 4)**

Given the predominance of male participants in your cohort, please acknowledge this as a potential limitation for generalizability and consider implementing gender-stratified analyses if statistically feasible.

**Response:** The following text was added to the limitations:

**Added Line 283-285:** “Additionally, our study population (Table 1) was predominantly older men. These results may not be generalizable to other non-veteran populations, younger patients, or women.”

Gender stratified analysis was presented in Table 3.

**5)Tables 2 and 4:** Please explicitly enumerate all confounding variables included in your statistical models as footnotes beneath the respective tables.

**Response:** Table footnotes have been changed to reflect what was adjusted for:

“Adjusted for age, race, ethnicity, sex, BMI, smoking, kidney disease, COPD, diabetes, hypertension, hyperlipidemia, obesity, chronic lung disease, PTSD, alcohol dependence, drug dependence, exposure to toxic substances, other viral infections, and immunodeficiency.”

**Discussion**

**1) COVID-19 Exposure and Cancer Risk**
The proposed immune-mediated reduction in cancer risk appears speculative and insufficiently developed. Please expand this critical section with a more comprehensive mechanistic discussion grounded in current immunological literature, while clearly delineating the hypothetical nature of these postulated mechanisms.

**Response:** The following was added to the Discussion section of the manuscript to address this comment:

**Revised and Added Lines 191 – 208:** “Our findings show that beyond the age of 45, exposure to COVID-19 is associated with a reduction in cancer incidence across all major cancer types. It is plausible that the immunologic changes that occur during acute COVID-19 infection (12, 13) might lead to the activation of monocytes with anticancer properties, as has been reported by other investigators (9). SARS-CoV-2 antigens induced increased expression of HLA-DR and TLR7 on monocytes compared to healthy cell controls (12). In the context of cancer, HLA-DR and TLR7 play distinct but interrelated roles. HLA-DR, a class II MHC molecule, is involved in antigen presentation to CD4+ T cells, a crucial process for mounting an adaptive immune response against cancer. TLR7, a Toll-like receptor, is involved in recognizing single-stranded RNA, which can be found in viral infections and also in some cancer cells (13). TLR7 can activate innate immune responses, potentially negatively influencing the tumor microenvironment and cancer cell behavior (14).The inflammatory response to COVID-19 may have a short-term protective benefit that wanes over time. Therefore, it is uncertain how long this risk reduction might be observed in the long term beyond the 3-year period of this study. Despite these possible mechanistic changes as a result of COVID-19 exposure, further research is required to explore the immunologic and physiologic properties of this potential association beyond those proposed in this paper. Care should be taken when interpreting these results, as these mechanisms are hypothetical and further definitive research is required.”

**2) Pandemic Healthcare Disruptions**
Your follow-up period encompasses up to 3 years post-infection. Please precisely delineate what proportion of this observation period coincides with the normalization of cancer screening practices, and provide appropriate contextual evidence or references supporting this temporal framework.

**Response**: The section was updated as follows:

**Revised and Added Lines 211-229:** “COVID-19 has introduced several new healthcare disruptions in cancer surveillance. Cancer trends during the pandemic showed an initial reduction, followed by a general return to the pre-pandemic levels (14, 15). One study looking at cancer screening rates among breast, cervical, and colorectal cancers found that screening initially dropped off during the start of the pandemic but resumed by the end of 2020, before experiencing a decline in 2021(16). An additional study showed that cancer incidence rates returned to their pre-pandemic trends in 2021, but those numbers did not fully incorporate delayed diagnosis from 2020 (17). These changes were expected, given the drastic reduction in preventive services in the US (18). An analysis of trends in US cancer rates revealed the highest variability in breast, cervical, prostate, and colorectal cancers, likely driven by a reduction in surveillance screening (19). By 2023, breast and colorectal screening had improved from the pandemic-related declines, with cervical cancer screening remaining below pre-pandemic levels (20). Additionally, the study collected data on all cancer types beyond those that are routinely screened for in clinical practice. It is uncertain how changes in cancer screening as described above, impacted the detection and treatment of these other cancer types. For this study, patients were followed for three years after the initial index infection. This included periods described above where the rates of screening for some cancer types returned to pre-pandemic levels or remained below expectations. Because both cohorts were exposed to similar screening delays and recovery over this period, it is unlikely that the difference in cancer rates between these groups could be attributed to this factor.”

**3) Age and Racial Differences:**

Given the distinctive attributes of the veteran population (regarding demographics, comorbidity profiles, and service history), please elaborate on how these characteristics might influence the external validity and generalizability of your findings to non-veteran populations.

**Response**: The following was added to the Discussion section:

**Added Line 283 – 285:** “our study population (Table 1) was predominantly comprised of older men. These results may not be generalizable to other non-Veteran population, younger patients, or women.”

**4)Age and Racial Differences line 173-176:**

The exclusion of individuals who succumbed within 30 days likely eliminates subjects with severe COVID-19 manifestations or compromised baseline health status. Would it be feasible to further stratify your cohort by COVID-19 severity indicators, such as hospitalization requirements or respiratory support needs, to explore differential cancer risk patterns?

**Response**: We agreed with this suggestion and further stratified out cohort by COVID-19 severity. We added accordingly to Methods, Results, Discussion, and Table 5 with a breakdown by mild, moderate, and severe cases.

The following was added to the Covariates sub-section of the paper:

Line 90-92: “COVID-19 severity was defined as following: mild as no hospitalization; moderate as hospitalized without ICU admission of high-flow oxygen; and severe as at least one of the following, high flow oxygen, intubation, mechanical ventilation, vasopressor, dialysis, ECMO.”

The following was added to the Results section:

Line 163-169: “Association of COVID-19 Infection and Decreased Cancer Risk by COVID-19 Severity.

When patients were stratified to three group according to COVID-19 severity, we showed that the reduction of newly diagnosed cancer risk was the most predominant in patients with mild COVID-19 (aHR=0.72, 95% CI: 0.70-0.74) (Table 5). In patients with moderate COVID-19, the reduced cancer hazard was 11% (aHR=0.89, 95% CI: 0.83-0.93). In patients with severe COVID-19, we did not show reduction in cancer hazard.”

The following section was added to the Discussion section of the paper:

**Added lines 257 – 269:** “

Impact of COVID-19 Severity

Our study attempted to separate cancer risk based by COVID-19 severity of mild, moderate, and severe. Interestingly, patients with mild disease had the highest benefit, followed by those with moderate disease. No benefit was observed in patients with severe disease. Given that one of the proposed mechanisms is immunologic changes induced by the COVID-19 ssRNA(9), patients with severe disease may have experienced a higher dysregulated immunological response compared to milder disease presentations (26), possibly driven by higher levels of dysfunction between the adaptive and innate immune systems in severe cases of COVID-19 (27). These changes in immunological response, which may prompt more severe disease progression, may reduce or eliminate any potential immunological changes that could aid in cancer surveillance. Further research is needed to investigate the degree of immune dysregulation in relation to COVID-19 severity and the development of inducible monocytes with cancer surveillance properties.”

|  | **Number of events (%)** | **aHR (95% CI)** | **C-Index(se)** |
| --- | --- | --- | --- |
| COVID-19 negative | 47614 (11.7%) | Reference | N/A |
| Mild | 5831 (7.8%) | 0.72 (0.70-0.74) | 0.735 (0.001) |
| Moderate | 1079 (12.6%) | 0.89 (0.83-0.93) | 0.729 (0.001) |
| Severe | 329 (11.9%) | 0.92 (0.82-1.02) | 0.729 (0.001) |

**Table 5.** aHR for New Cancer Diagnosis within three years for Patients with Positive COVID-19 Testing Compared to Patients of Negative Testing by Severity. Adjusted for age, race, ethnicity, sex, BMI, smoking, kidney disease, COPD, diabetes, hypertension, hyperlipidemia, obesity, chronic lung disease, PTSD, alcohol dependence, drug dependence, exposure to toxic substances, other viral infections, and immunodeficiency
